# Development of a core SNP arrays based on the KASP method for molecular breeding of rice

**DOI:** 10.1186/s12284-019-0272-3

**Published:** 2019-04-08

**Authors:** Guili Yang, Siping Chen, Likai Chen, Kai Sun, Cuihong Huang, Danhua Zhou, Yuting Huang, Jiafeng Wang, Yongzhu Liu, Hui Wang, Zhiqiang Chen, Tao Guo

**Affiliations:** National Engineering Research Center of Plant Space Breeding, South China Agricultural University, Guangzhou, 510642 China

**Keywords:** Rice, KASP, Core SNP array, Molecular breeding

## Abstract

**Background:**

The development and utilization of genetic markers play a pivotal role in marker-assisted breeding of rice cultivars during pyramiding of valuable genes. Among molecular markers, SNPs have become the most promising due to their wide distribution within genomes and suitability for high -throughput automated genotyping. Although metadata of SNPs have been identified via next generation sequencing in rice, a large gap between the development of SNP markers and the application in breeding still exists. To promote the application of SNP markers based on the KASP (Kompetitive Allele-Specific PCR) method in rice breeding, a set of core SNP arrays was built via the screening of SNP databases and literature resources based on the KASP method.

**Results:**

Five hundred and ninety six SNPs classified into eight subsets including quality control, indica-indica variation, highly polymorphic, functional genes, key genes targeting sites, gene cloned region, important trait associated and gap filling sites were chosen to design KASP primers and 565 out of them were successfully designed, and the assay design success rate was 94.8%. Finally, 467 out of the 565 successfully-designed SNPs can display diversity at the loci were used to develop a set of core SNP arrays. To evaluate the application value of the core SNP markers in rice breeding, 481 rice germplasms were genotyped with three functional KASP markers designed from the sequences of *GBSSI*, *SSIIa*, and *Badh2* from the core SNP arrays for estimation of their grain quality performance. Eighteen rice lines, including Xiangwanxian 13, Basmati 370, Ruanhua A, and PR 33319–9–1-1-5-3-5-4-1, harbor all three favorable alleles. The core KASP arrays were also used for rice germplasm assessment, genetic diversity and population evaluation. Four hundred and eighty-one rice germplasms were divided into 3 groups: POP1, POP2 and POP3. POP1 and POP2 were indica rice subgroups consisting of 263 and 186 rice germplasms, respectively. POP3 was a japonica rice subgroup consisting of 32 rice germplasms. The average *F*_*ST*_ value for the three subgroups was 0.3501; the *F*_*ST*_ value of POP1 and POP3 was the largest (0.5482), while that of POP1 and POP2 was the smallest (0.0721). The results showed that the genetic distance between the japonica and indica rice subspecies was large, indicating that the core SNP markers were effective at discriminating the population structure of the germplasms. Finally, the core KASP arrays were used for association analysis with milled grain traits. A total of 31 KASP markers were significantly associated (*P* < 0.01) with ML and the LWR. Among the 31 markers, 13 were developed based on cloned genes or on identified loci related to yield traits. Notably, several KASP markers associated with grain quality were also found to be associated with brown planthopper resistance or green leafhopper resistance simultaneously.

**Conclusions:**

The core KASP arrays developed in our study were efficient and versatile for rice germplasm assessment, genetic diversity and population evaluation and are valuable for promoting SNP molecular breeding in rice. Our study demonstrated that useful assays combined with molecular breeding can be exploited for important economic trait improvement in rice breeding.

**Electronic supplementary material:**

The online version of this article (10.1186/s12284-019-0272-3) contains supplementary material, which is available to authorized users.

## Background

Cultivated rice (*Oryza sativa* L.) is one of the most important crops and supplies food to approximately half of the human global population (Chen et al. 2014). Molecular marker-assisted selection (MAS), combined with conventional breeding approaches, enables breeders to identify individual genotypes that are precisely associated with different economically important traits, which can dramatically improve rice breeding efficiency (Tian et al. 2010). The development and utilization of genetic markers play a pivotal role in marker-assisted breeding of rice cultivars during pyramiding of valuable genes. High polymorphism, codominant inheritance, high density, high throughput, and easy automation and data exchange are the characteristics of ideal DNA markers (Tripathi et al. 2016). In addition, making full use of cloned genes that control agronomic traits or highly reliable quantitative trait loci information to develop molecular markers that are closely related to traits is also an important for improving the efficiency of molecular breeding.

Since the discovery of single-nucleotide polymorphisms (SNPs) in 1994, SNPs have been widely used in many fields, such as biology, agriculture, medicine, and biological evolution. Among molecular markers, SNPs have become the most promising due to their wide distribution within genomes and suitability for high-throughput automated genotyping. High-throughput sequencing technology has greatly advanced the discovery of SNP markers. A total of 408,898 candidate DNA polymorphisms (SNPs and/or insertions/deletions (InDels) were identified via comparative analysis of the draft genome sequences of the rice cultivars Nipponbare (*japonica*) and 93–11 (*indica*) (Feltus et al. 2004) under the support of the International Rice Genome Sequencing Project 2005. One hundred sixty thousand nonredundant SNPs (McNally et al. 2009) were identified by sequencing 100 Mb of specific reference genome sequences of 20 different cultivars, including local cultivars. Since 2011, rice resequencing projects have also provided an abundance of information about rice SNP markers, and databases have been constructed for querying and determining rice SNP loci, e.g., the Gramene database (http://ensembl.gramene.org/genome_browser/index.html), the Rice Diversity Project database (https://ricediversity.org/), the Rice Genome Annotation Project database (http://rice.plantbiology.msu.edu/), and the Rice SNP-Seek Database (http://snp-seek.irri.org). In 2015, SNPs and small-fragment InDels of 2859 rice genomes of good quality were obtained via the genome-wide resequencing of 3000 rice resources worldwide (Alexandrov et al. 2015). These genomic variation data were used to compile a comprehensive SNP and InDel polymorphism sub-database for rice functional genomics breeding. Moreover, in recent years, via quantitative trait loci (QTL) mapping, gene mapping, and genome-wide association studies (GWASs), researchers have also discovered a large number of genetic loci that contribute to important agronomic traits in rice. More than 800 loci associated with development, yield, quality, and biotic and abiotic stress have been cloned or finely mapped (Jiang et al. 2012, Jiang et al. 2012, Chen et al. 2013). These reported SNPs, genes and genetic loci information provide a reliable basis for the application of SNP markers in rice molecular breeding.

However, a large gap between the development of SNP markers and the application of rice breeding still exists. First, the current SNP databases provide a large amount of SNP information, including redundant information, and it is difficult for breeders to quickly obtain useful SNPs. For example, the Rice 3 K project provides more than 18 million different types of SNP information; this amount actually exceeds that possible for breeders to analyze. Therefore, it is important that the classification of core SNPs represents key information. Second, the relationships between a large number of SNPs and reported genes or genetic loci are not clear. There is a chronic shortage of functional, diagnostic or tightly linked SNP markers for use in molecular breeding. Currently, only a few diagnostic SNP markers are used in rice breeding, e.g., *Wx*, *ALK*, *Pikh*, *GS3*, *GW5*, and *CHALK5*, and it is difficult to meet the needs of molecular breeding to improve more important agronomic traits. Third, the methods of SNP detection are not flexible and adaptable to the changing demands of end-users. For example, hybrid-based SNP chips can accommodate a large amount of locus genotyping at one time, but the cost of detection is high; in addition, it is difficult to analyze the genotypes of various individual plants derived from genetically separate populations. Cleaved amplified polymorphic sequence (CAPS) markers can also be used for SNP allelic genotyping (Chen et al. 2009), but producing high-throughput genotyping results is difficult because of the inefficiency of the enzyme digestion system. The high-resolution melting (HRM) method based on DNA melting temperature analysis to detect SNP alleles strongly depends on complex multiplex PCR, which can easily lead to false positives. Sequencing is the most direct and accurate method for SNP detection, but it is expensive and requires professional bioinformatics analysis. Therefore, continued development of core SNP markers that are both closely related to agronomic traits and suitable for different needs is highly valuable.

Kompetitive Allele-Specific PCR (KASP) is a detection method that can type SNPs and InDels at specific sites. With respect to genotyping, KASP is based on terminal fluorescence reading, similar to the basis of TaqMan probe detection. Each method uses two-color fluorescence to detect individual samples of different genotypes at a single site; different fluorescent products reflect different DNA templates. For KASP tests, a SNP- or Indel-specific KASP assay mixture (specific primer mixture), KASP Master Mix (general mixture) and DNA samples are mixed together for thermal cycling, after which the fluorescence is read by laser scanning at the end point. KASP-SNP markers are highly accurate, inexpensive and highly flexible, and they have a high conversion rate and a wide range of applications. Several functional SNPs and InDels have been converted into functional markers (FMs) via KASP assays, greatly improving the speed and efficiency of selection in crop breeding programs (Neelam et al. 2013, Pariasca-Tanaka et al. 2015, Rasheed et al. 2016). Using the KASP method, researchers from the International Rice Research Institute (IRRI) have developed a total of 2015 SNP markers distributed throughout the genome for use in analyzing the genetic backgrounds of rice strains (Pariasca-Tanaka et al. 2015). However, few KASP-SNP functional or diagnostic markers have been reported, and classification of SNP arrays of high polymorphism and agronomic-associated traits is lacking. These factors are limiting the application of KASP-SNP markers in breeding procedures.

To promote the application of KASP-SNP markers in rice breeding, in the present study, eight subsets of SNP information were obtained by querying international rice genome databases and literature resources indica-indica variation, highly polymorphic, functional genes, key genes targeting sites, gene cloned region, important trait associated and gap filling sites. These SNP loci were converted into KASP markers, and 530 rice germplasms were genotyped with these SNP markers. Based on the results of genotyping, a set of core SNPs with high reliability was identified. To evaluate the application value of these core SNP markers in rice breeding, the population structure, allelic functional gene variation and correlations between SNP markers and grain characteristics were analyzed with the use of the identified core SNP markers. The findings of this study are valuable for promoting SNP molecular breeding in rice.

## Results

### Conversion rate and genetic diversity of KASP marker assays

**Fig. 1:**
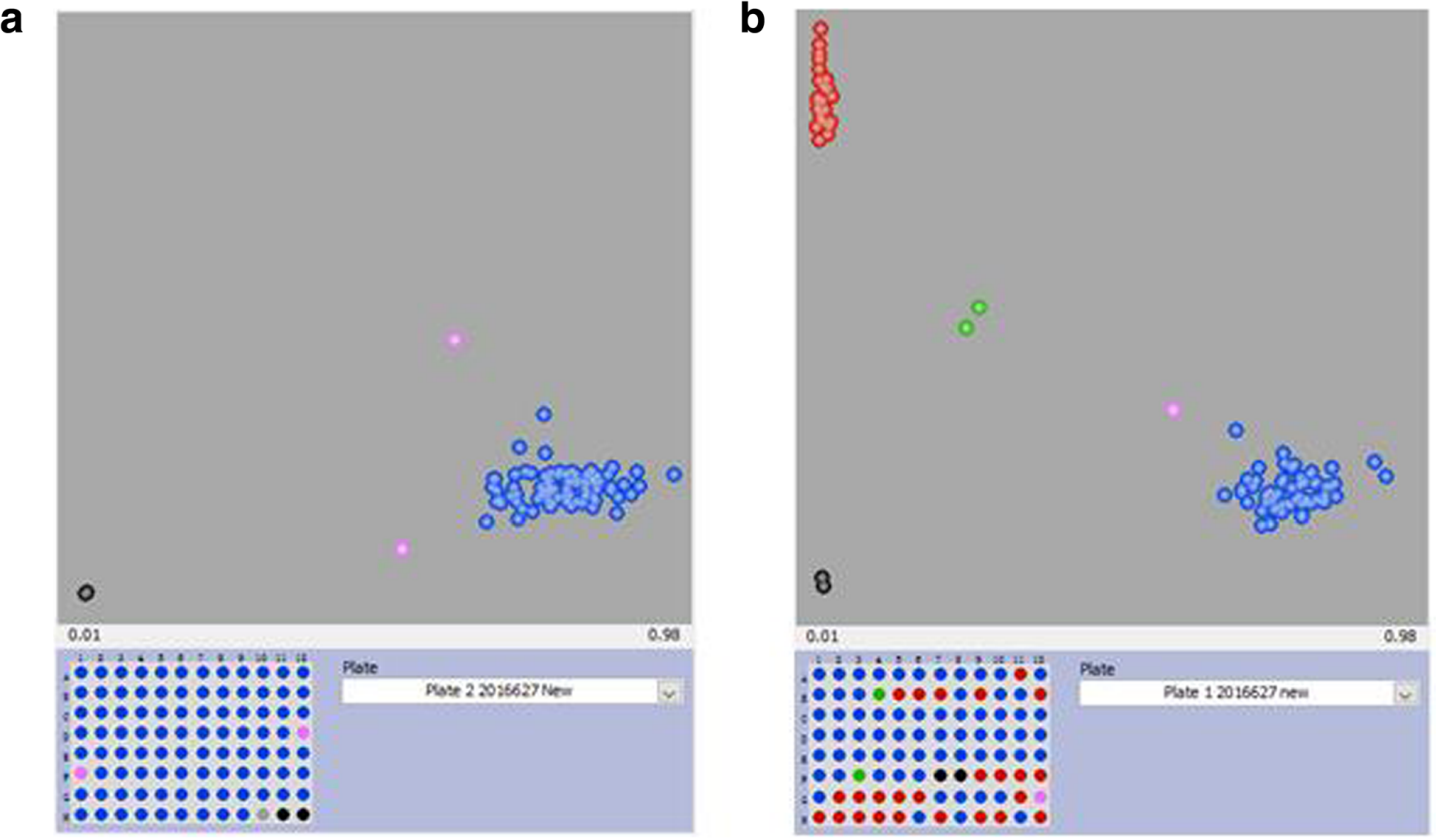
Valid and invalid markers. **a** invalid markers generate one fluorescent signal; **b** valid markers generate at least t effective fluorescent signals

**Fig. 2:**
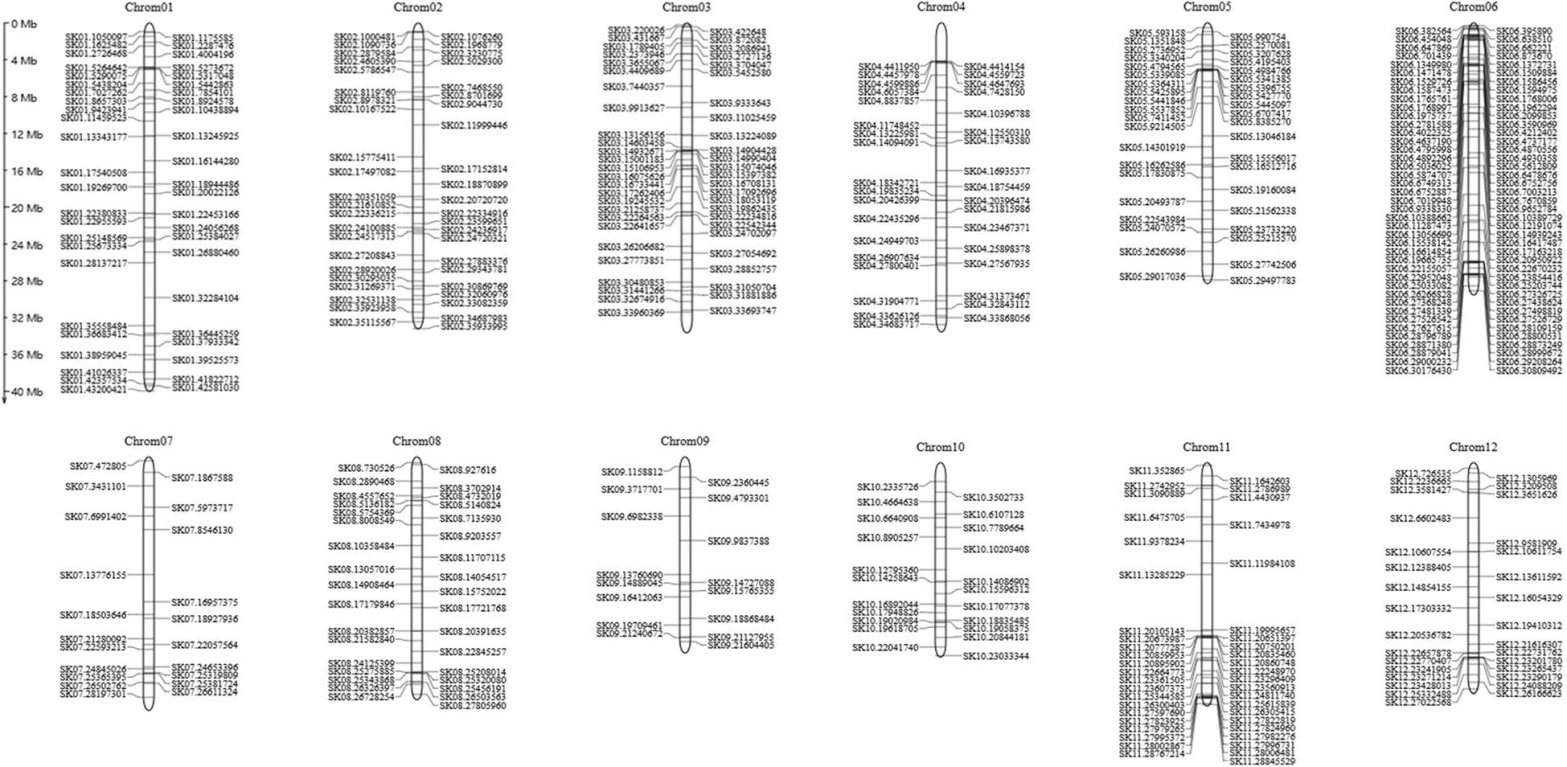
Distribution of KASP-SNP markers among rice chromosomes

In the present study, KASP primers were designed from 565 out of 596 SNP sites successfully, and the assay design success rate was 94.8% (Table 1). The genotypes of 530 rice accessions were identified using the designed KASP primers. Among the 565 KASP markers, 98 were discarded because they could generate only one genotype call, thus displaying no diversity at the loci (Fig. 1a). A total of 467 markers showed at least 2 effective fluorescence signals; these SNPs were used to develop core KASP-SNP assays in our study (Fig. 1b). We analyzed the distribution of all the selected SNPs within the genome (Table 2). In general, the KASP markers were distributed relatively evenly, with an average density of 1.24 SNPs/Mb throughout all chromosomes, with the exception of chromosome 6. For visual viewing, a physical map of these markers was constructed via the mapping website Map Gene 2 Chromosome V2 (Fig. 2).

**Table 1:** Candidate SNPs chosen for KASP assays

Subset	Characters of SNPs	No. of SNPs	No. of assay successfully-designed	No. of core SNPs	Assay design success rate
1	Subgrouping or quality control related	61	59	57	96.7
2	Indica/Indica variation related	64	62	60	96.8
3	SNPs with high polymorphism information content (PIC)	53	52	51	98.1
4	Functional nucleotide polymorphisms (FNPs)	21	18	14	85.7
5	Targeting site of cloned genes	95	90	86	94.7
6	SNPs overlapped gene interval	173	156	113	90.1
7	Reliable SNP in GWAS	33	32	31	97.0
8	Gap filling SNP	96	96	55	100.0
	Total	596	565	467	94.8

**Table 2:** Distribution of valid KASP markers within each chromosome

Chr.	No.	Chr. length/Mb	Marker density(SNP/Mb)
1	47	43.27	1.09
2	44	35.94	1.22
3	51	36.41	1.40
4	35	35.50	0.99
5	40	29.96	1.34
6	82	31.25	2.62
7	21	29.70	0.71
8	34	28.44	1.20
9	16	23.01	0.70
10	22	23.21	0.95
11	44	29.02	1.52
12	31	27.53	1.13
Average	39	31.10	1.24
Total	467	373.25	

**Fig. 3:**
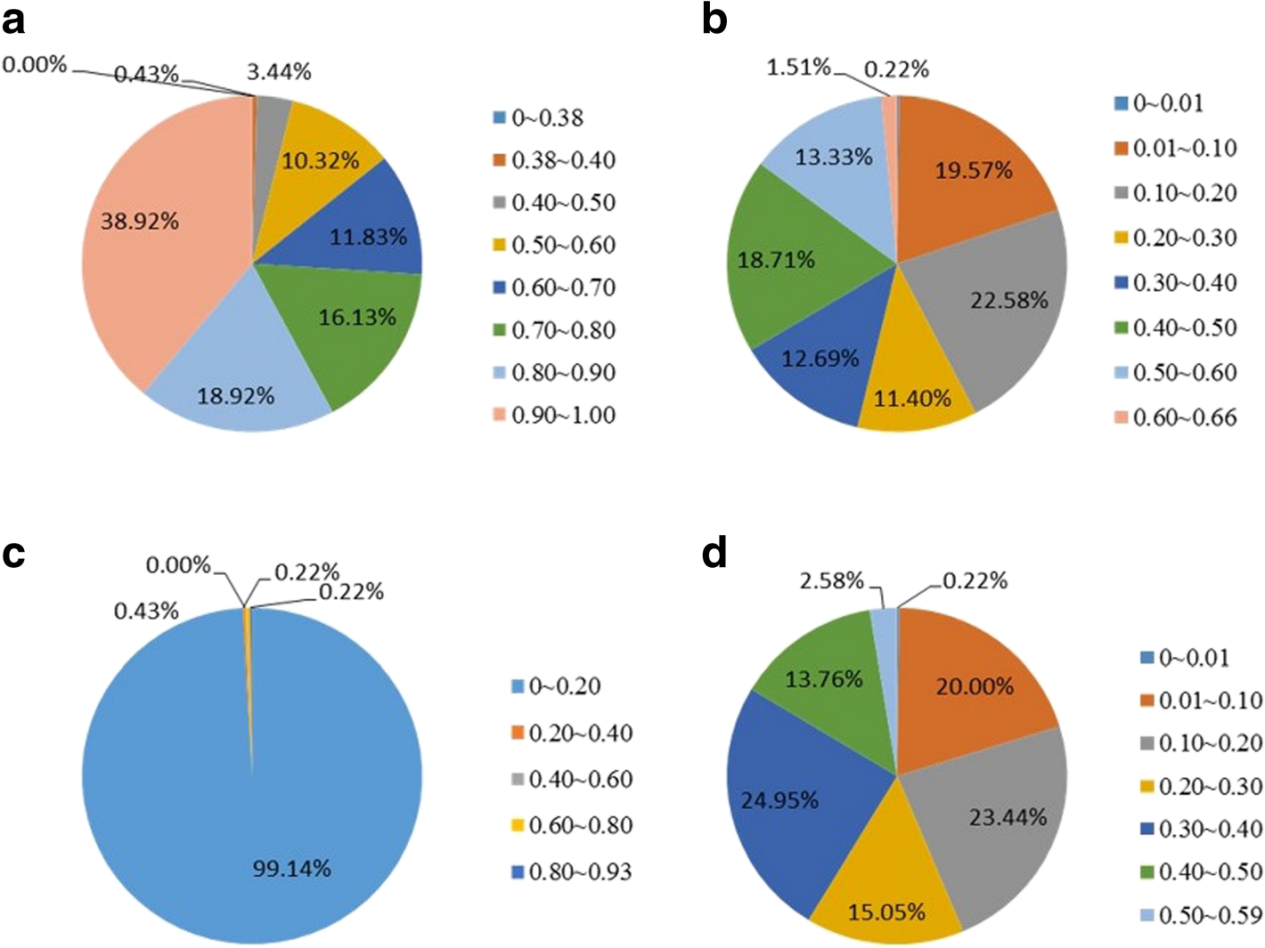
Genetic diversity of 467 KASP markers **a**: Minor allele frenquency; **b**: Gene diversity; **c**: Heterozygosity; **d**: Polymorphism information content

Marker assessment analysis revealed that the mean frequency, gene diversity, heterozygosity and polymorphism values were 0.80, 0.28, 0.01, and 0.24, respectively. Detailed analysis revealed that relatively high levels of polymorphism (measured by the minor allele frequency (MAF)) in the test range of rice accessions ranged mainly from 0.50 to 1.00 (Fig. 3a). The gene diversity ranged mainly from 0.01 to 0.60, with an average gene diversity of 0.28, and the heterozygosity across all loci ranged mainly from 0.00 to 0.93 (Fig. 3b,c). The PIC value ranged from 0.01 to 0.50, with an average value of 0.24 (Fig. 3d). Forty rare alleles (whose gene frequencies were less than 1%) were detected in 50 rice plants at 40 sites; these alleles accounted for 4.3% of all alleles. These rare alleles were distributed in 50 rice lines, 26 of which were collected from IRRI varieties, indicating the specific variation that exists within IRRI germplasms.

### Classification of core SNP markers associated with agronomic traits

According to the physical position of the cloned gene or GWAS-identified locus, 244 markers were found to be related to important rice traits (yield, quality, resistance, root development, plant development and fertility) (Table 3). Yield-related markers were distributed across all the chromosomes, and quality-related markers were distributed mostly on chromosome 6. Markers related to plant development were distributed on chromosomes 1, 2, 3, 6, 7 and 11, and two fertility-related markers were located on chromosome 10. Of these 244 markers, 14 were developed based on the functional loci of cloned genes and could specifically distinguish between the functional alleles, 86 originated from polymorphic loci of the coding sequences of cloned genes and were considered gene targeting markers that can be used to track target genes, and 113 were considered gene overlapping markers because they originated from interval regions of cloned genes. In addition, 31 highly reliable trait-related SNP loci (based on previous GWAS results) were used to develop to KASP markers.

**Table 3:** Distribution of KASP markers associated with agronomic traits

	Yield	Quality	Resistance	Fertility	Development	Heading	Total
Chr1	16	1	1	/	2	1	21
Chr2	13	1	1	/	8	1	24
Chr3	12	1	9	/	12	/	34
Chr4	2	3	7	/	/	/	12
Chr5	15	2	1	/	/	/	18
Chr6	14	13	31	/	1	5	64
Chr7	3	2	/	/	2	/	7
Chr8	5	5	2	/	/	/	12
Chr9	4	/	/	/	/	/	4
Chr10	/	2	1	2	/	1	6
Chr11	3	/	24	/	1	/	28
Chr12	/	1	13	/	/	/	14
total	87	31	90	2	26	8	244

Notably, 14 functional SNP markers were characterized, including those related to grain quality, blast resistance, fertility and grain shape (Table 4). These functional SNP markers are responsible for corresponding traits and have significant value for superior allele screening and molecular breeding. Among these FMs, *Wx*, *ALK* and *fgr* can be used to improve eating quality, and *Rf4* plays an important role in the molecular breeding of hybrid rice parents. Two dominant R genes, *Pik* and *Pi2*, were thought to provide broad-spectrum and high resistance to blast, so the pyramid of these two genes is valuable for breeding rice varieties that exhibit robust blast resistance. Two KASP-SNP markers, SK11.27979265 and SK06.10389729, were developed to distinguish between alleles of *Pik* and *Pi2*. *GS3*, a main QTL controlling grain weight and grain length, accounted for 80–90% of the variation in grain weight and grain length in a recombinant inbred line (RIL) population (Fan et al. 2006). On the basis of the A/C allele of exon 2 of the *GS3* gene, the SNP marker SK03.16733441 was developed to distinguish between AA and CC variants, of which the AA allele is responsible for long-grain phenotypes.

**Table 4:** Functional KASP markers

Marker	Chromosome	Physical location	Polymorphic type	Gene	Trait
SK06.1765761	6	1,765,761	G/T	*GBSSI/Wx*	amylose content
SK06.1768006	6	1,768,006	A/C	*GBSSI/Wx*	amylose content
SK06.1768997	6	1,768,997	C/T	*GBSSI/Wx*	amylose content
SK06.6752887	6	6,752,887	GC/TT	*SSIIa/ALK*	gelatinization temperature
SK06.6752756	6	6,752,756	A/G	*SSIIa/ALK*	gelatinization temperature
SK08.20382857	8	20,382,857	AAAAGATTATGGC/TATAT	*Badh2/fgr*	grain fragrant
SK08.8008549	8	8,008,549	A/C	*fgr*	grain fragrant
SK08.5140824	8	5,140,824	C/T	GPT1	amylose content
SK12.10607554	12	10,607,554	G/T	*Pita*	blast resistance
SK05.3340204	5	3,340,204	A/T	*Chalk5*	chalkiness
SK10.18835485	10	18,835,485	A/C	*Rf4*	fertility
SK11.27979265	11	27,979,265	G/T	*Pik*	blast resistance
SK06.10389729	6	10,389,729	CAGGAAT/TGTTATT	*Pi2*	blast resistance
SK03.16733441	3	16,733,441	A/C	*GS3*	grain length

### Confirmation of KASP-SNP results via sanger sequencing

To evaluate the accuracy of the KASP-SNP genotyping results, Sanger sequencing and KASP genotyping primers were designed for the *Pita* gene. *Pita* is a blast resistance gene, and there is only one amino acid difference in the proteins of the two alleles encoded by the *Pita* locus; i.e., the 918th amino acid changes from alanine to serine, which is responsible for the change in resistance to susceptible. One SNP causes this amino acid difference: genotype G/G represents resistance, while T/T confers susceptibility. KASP genotyping was performed on 11 materials whose *Pita* genotypes are known, and the genotyping results were compared with those of Sanger sequencing. In terms of primer design, Sanger sequencing primers, Pita-F and Pita-R, were designed for amplifying 300 bp length sequences, which include SNP loci (Additional file 1: Table S1). Three primers were used for KASP genotyping: Pita-F, Pita-H and Pita-C. The first two primers accompany the FAM or HEX fluorescent markers, while Pita-C is a universal primer (Additional file 1: Table S1).

**Fig. 4:**
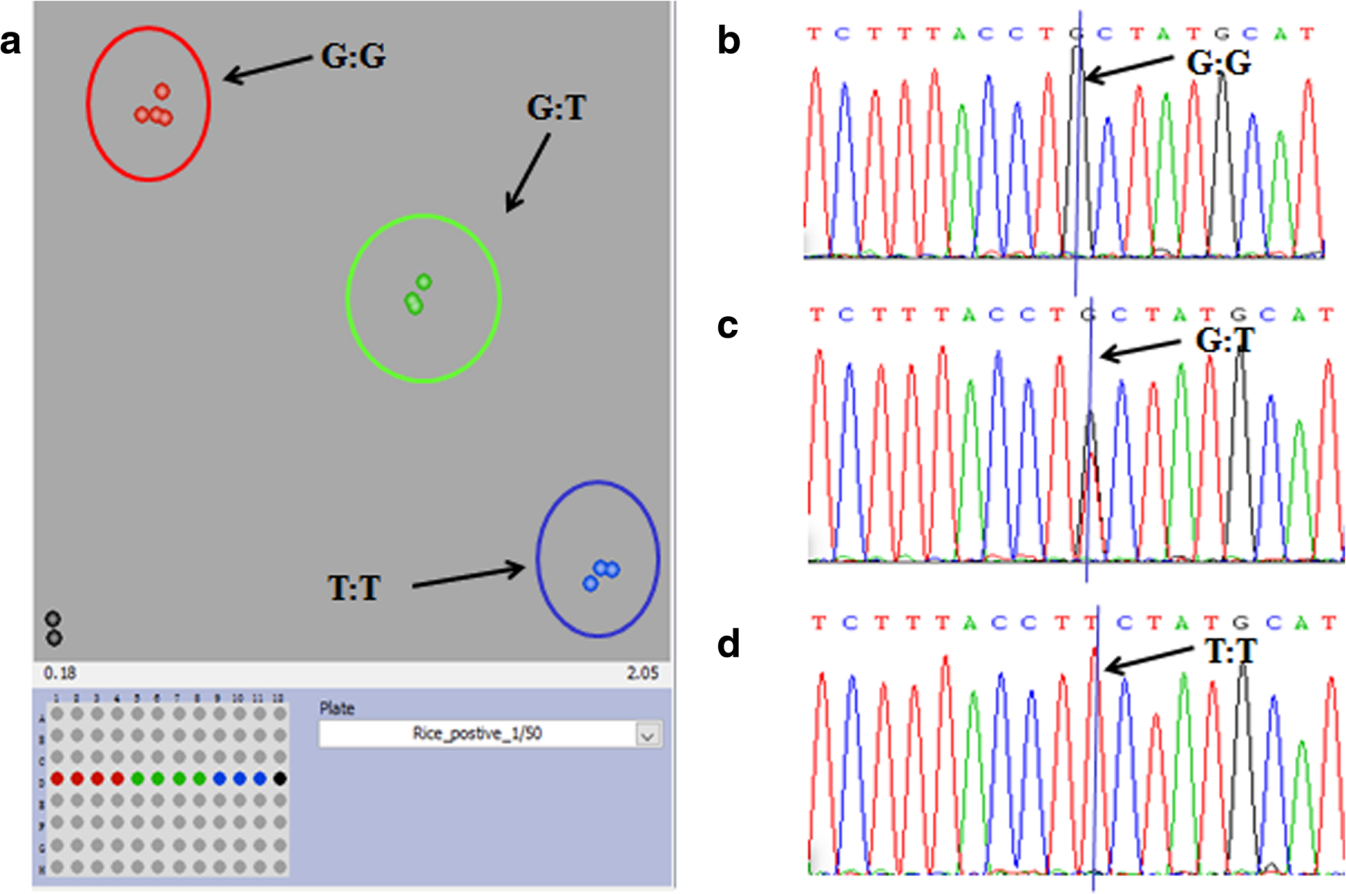
Results of KASP genotyping and Sanger sequencing. **a** KASP genotyping of *Pita*; **b** the sequencing peak of the G/G allele; **c** the sequencing peak of the G/T allele; **d** the sequencing peak of the T/T allele

KASP primers were used to investigate 11 materials whose genotypes are known. The resistance group consisted of C296, C297, C298 and C304, all of which harbor the GG allele, while the susceptible group, comprising C258, C259 and C260, harbor the TT allele. The heterozygous group, which consisted of C275, C276, C277 and C278, had G/T alleles. The three groups were divided into different clusters according to fluorescence, as shown in Fig. 4a. The results are consistent with those of Sanger sequencing: genotypes G/G and T/T exhibited a single peak, while the heterozygous T/G genotype exhibited a double peak (Fig. 4b, c, d).

### Application of core SNP markers for rice germplasm assessment, genetic diversity and population evaluation

**Fig. 5:**
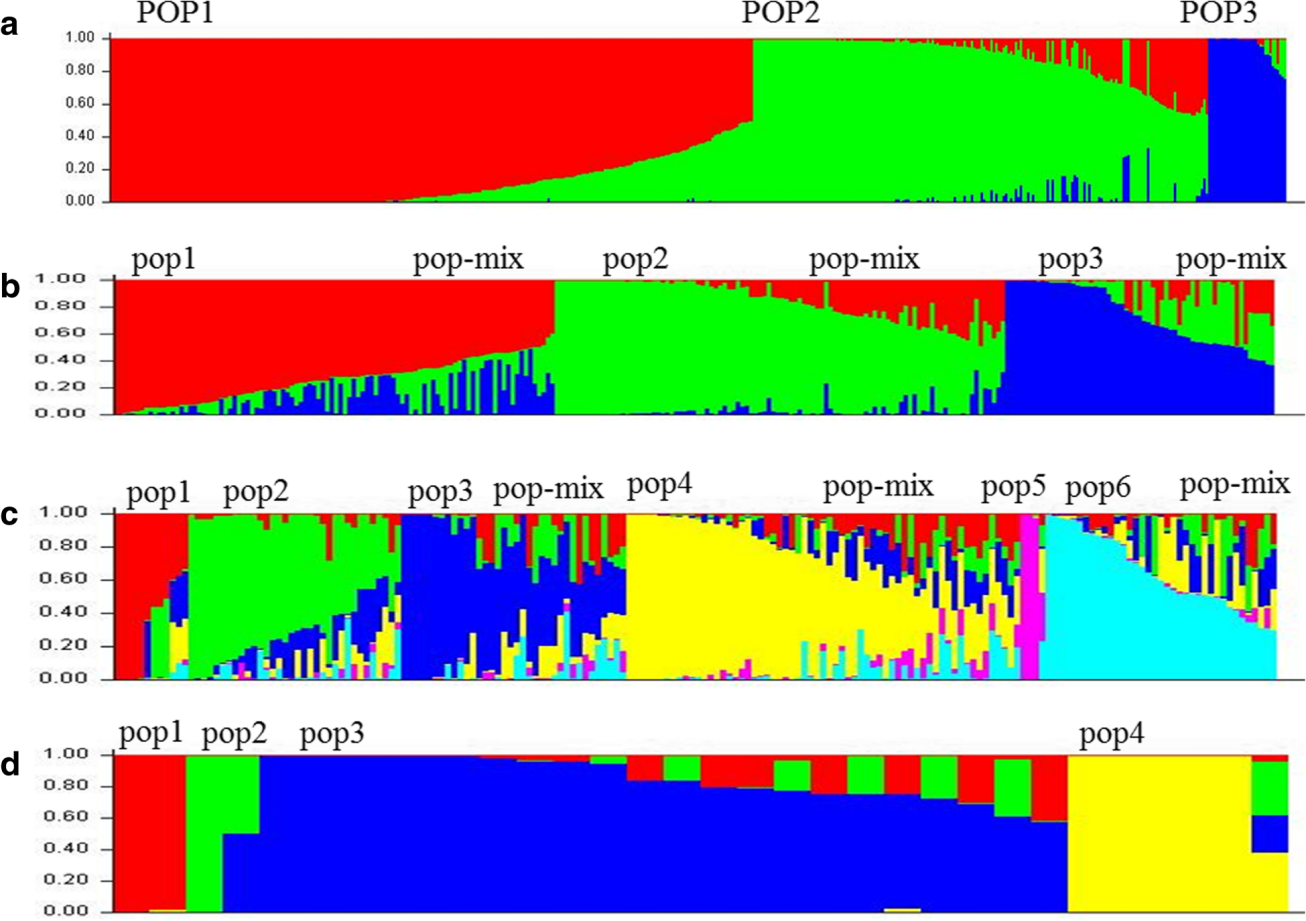
Population Structure of 3 groups. **a** Four hundred eighty-one rice germplasms were divided into 3 groups: POP1, POP2 and POP3; **b** the subgroups of POP1; **c** the subgroups of POP2; **d** the subgroups of POP3

Based on the genotyping results of 467 core SNP markers, the population structure of 481 rice germplasms was classified by Structure software. The population number K was set to 2–11, and each hypothetical K value was calculated five times. The ΔK of Evanno was maximal at K = 3. Therefore, the 481 rice germplasms were divided into 3 groups: POP1, POP2 and POP3 (Fig. 5a). POP3 was a japonica rice subgroup consisting of 32 rice germplasms. POP1 and POP2 were indica rice subgroups consisting of 263 and 186 rice germplasms, respectively. The F statistic (*F*_*ST*_ value) of the populations could strongly explain the genetic distance between populations. The average *F*_*ST*_ value for the three subgroups was 0.3501; the *F*_*ST*_ value of POP1 and POP3 was the largest (0.5482), while that of POP1 and POP2 was the smallest (0.0721). The results showed that the genetic distance between the japonica and indica rice subspecies was large, indicating that the core SNP markers were effective at discriminating the population structure of the germplasms.

**Fig. 6:**
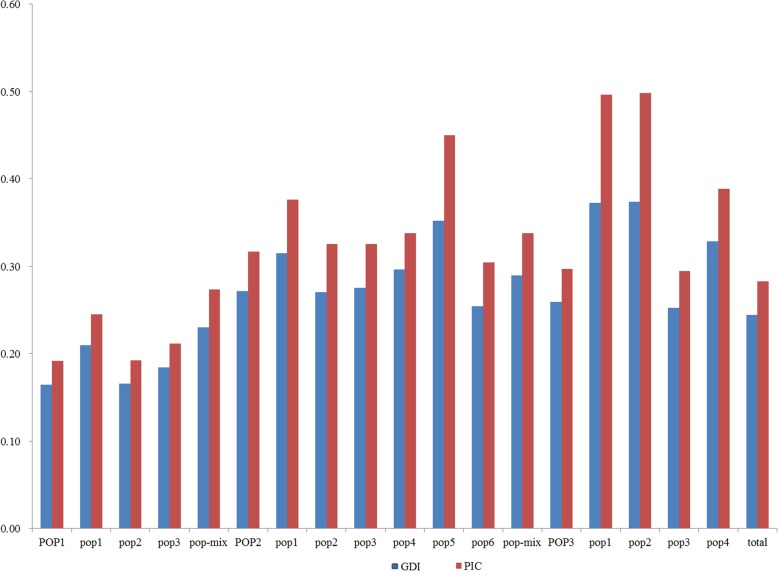
Comparison between the GDI and PIC among subgroups. GDI: gene diversity index; PIC: polymorphism information content

POP1 was further divided into pop1, pop2, pop3 and pop-mix subgroups, which include 92, 94, 52 and 25 rice germplasms, respectively (Fig. 5b). POP2 was divided into 7 subgroups, pop1, pop2, pop3, pop4, pop5, pop6 and pop-mix, which consist of 8, 27, 26, 37, 3, 31 and 54 rice germplasms, respectively (Fig. 5c), and 4 subgroups were identified within POP3, pop1, pop2, pop3 and pop4, which include 2, 2, 23 and 6 rice germplasms, respectively (Fig. 5d). The average allele number per locus (ANP), the gene diversity index (GDI), and the PIC value of the POP1 population were 2.69, 0.19, and 0.16, respectively. The POP2 population exhibited high genetic diversity, with an ANP of 2.88, a GDI of 0.32 and a PIC of 0.27. For POP3, the ANP, GDI, and PIC were 2.53, 0.30, and 0.26, respectively (Table 5). These results revealed no significant correlation between the GDI and the number of materials in each subgroup, but there was a positive correlation between gene diversity and the PIC. The genetic diversity and PIC of each subgroup and corresponding group were essentially similar (Fig. 6). Based on the above results, we concluded that the core SNP markers have a superior ability to reflect the genetic diversity of different materials.

**Table 5:** Statistical results of the genetic diversity of the groups

Group	Subgroup	Number of materials	Average allele number of per locus (ANP)	Gene diversity index (GDI)	Polymorphic information content (PIC)
POP1		263	2.69	0.19	0.16
	pop1	92	2.63	0.25	0.21
	pop2	94	2.48	0.19	0.17
	pop3	52	2.55	0.21	0.18
	pop-mix	25	2.25	0.27	0.23
POP2		186	2.88	0.32	0.27
	pop1	8	2.25	0.38	0.31
	pop2	27	2.37	0.33	0.27
	pop3	26	2.48	0.33	0.28
	pop4	43	2.71	0.34	0.30
	pop5	3	2.03	0.45	0.35
	pop6	25	2.28	0.30	0.25
	pop-mix	54	2.79	0.34	0.29
POP3		32	2.53	0.30	0.26
	pop1	2	2.00	0.50	0.37
	pop2	2	2.00	0.50	0.37
	pop3	22	2.36	0.29	0.25
	pop4	6	2.24	0.39	0.33
total		481	2.98	0.28	0.24

**Fig. 7:**
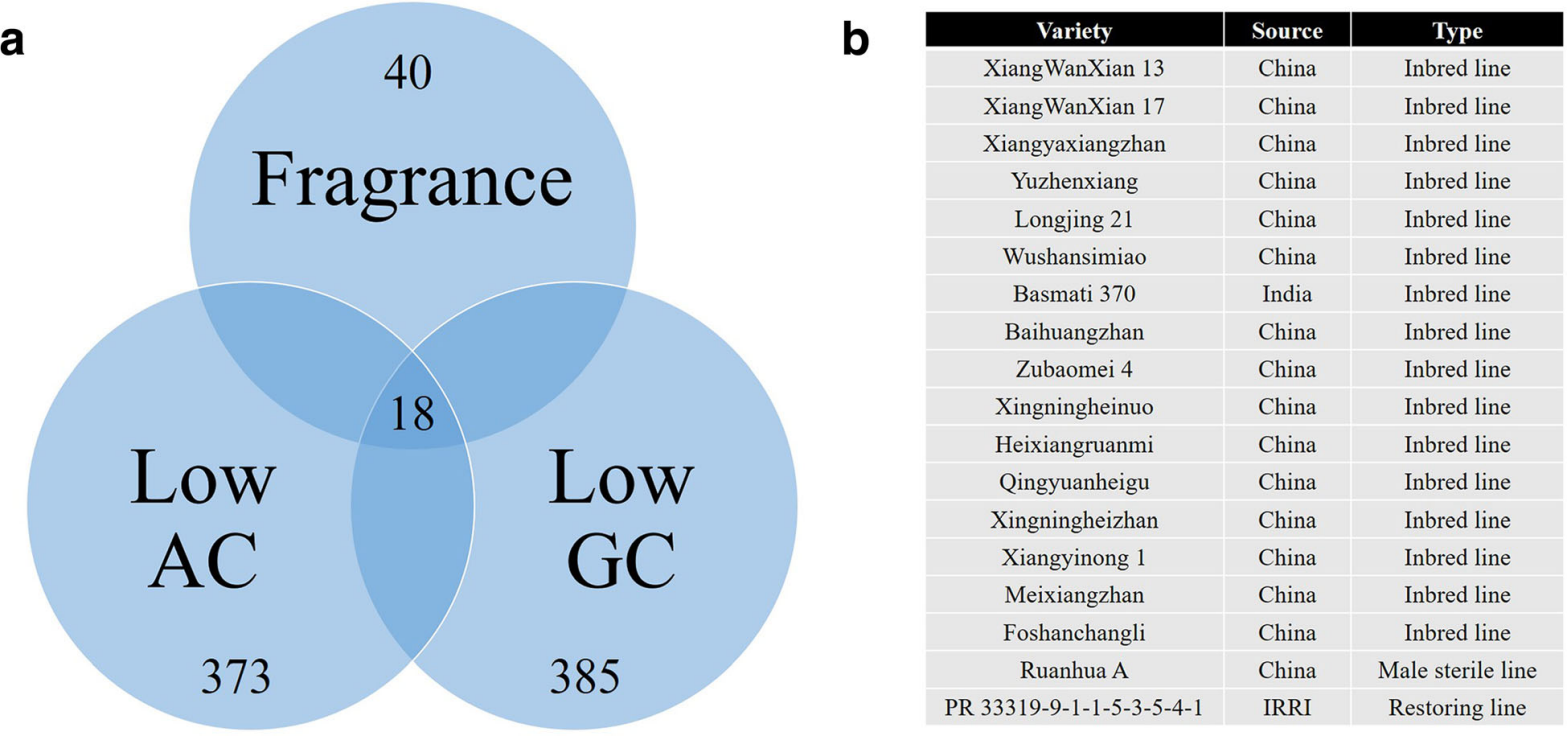
Screening results of favorable alleles. **a** Venn diagrams of rice lines with fragrance, low amylose content (AC) and low gel consistency (GC); **b** Eighteen rice lines harboring the three the favorable alleles of GBSSI (T allele), SSIIa (TT allele), and Badh2 (Del allele)

To utilize the benefits from the functional KASP markers used in our genotyping panel, molecular screening was carried out. Using grain quality properties as an example, the allelic types of three FM genes, *GBSSI*, *SSIIa*, and *Badh2*, were screened among the 481 rice germplasms. The results are shown in Fig. 7. In total, 385, 373, and 40 rice lines that harbor the favorable alleles of *GBSSI* (T allele), *SSIIa* (TT allele), and *Badh2* (Del allele) were identified, respectively (Fig. 7a). Eighteen rice lines, including Xiangwanxian 13, Basmati 370, Ruanhua A, and PR 33319–9–1-1-5-3-5-4-1, harbor all three favorable alleles (Fig. 7b). These functional KASP markers of the core SNP array constitute a convenient and helpful method for excavating elite rice strains for breeding.

### Utilization of the core SNP array to identify loci associated with milled grain traits via GWASs

In the core SNP array, 244 markers are related to important rice traits (yield, quality, biotic or abiotic stress, plant development and fertility, etc.). These markers provide important reference information for MAS breeding. To evaluate the validity of these trait-related markers, with milled grain properties as an example, a GWAS was conducted on 323 rice accessions via core SNP genotyping. We focused on the contribution of yield-related markers to grain traits and tried to identify new markers that affecting grain traits. Of the 244 markers, 18 were developed based on cloned genes related to grain shape or on reported GWAS-identified loci related to grain shape. Among these 18 markers, 9 were developed based on functional or polymorphic sites within the cloned genes *GS3* and *GW5*, and the other 9 were developed based on highly reliable GWAS-identified loci. In this study, we paid special attention to validating whether these markers could be detected repeatedly.

**Fig. 8:**
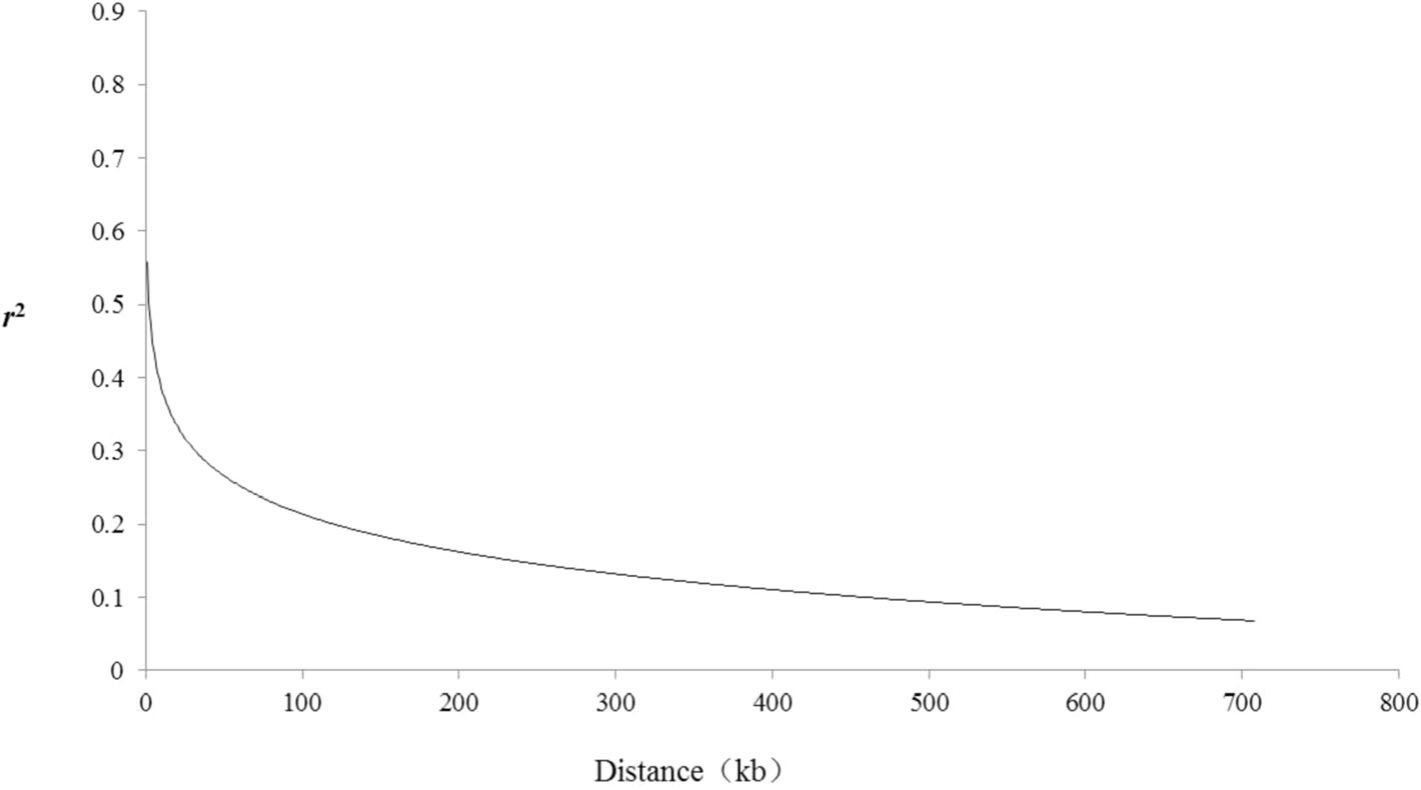
Genome-wide average LD decay estimated from 323 rice accessions

The genotypes of 323 rice accessions were obtained via the core SNP array, and the kinship of the accessions was evaluated via SPAGeDi_1-2 g software. Most of the accessions had kinship values that were less than 0.1, while only 2.73% of the materials had kinship values greater than 0.3. The kinship values indicated that the population accessions had a relatively low genetic relatedness and that the population was suitable for GWASs. A certain degree of linkage disequilibrium (LD) exists between the locus combinations of the core markers. The regression equations governing the R^2^ parameters and the physical distance of the LD paired sites were analyzed by Tassel 5.0. The resulting equation y = 0.075ln(x) + 0.558 reflects the relationship between the R^2^ values and the LD. When an R^2^ value decreases to half of the maximum value, the attenuation distance is approximately 100 kb, which is consistent with results in previous studies (Fig. 8). Marker-trait associations for milled grain length (ML), milled grain thickness (MT), the LWR, and milled grain weight (MRW) were evaluated via the program TASSEL. A total of 31 KASP markers were significantly associated (*P* < 0.01) with ML and the LWR (Table 6). Eighteen of 31 markers were associated with more than two traits; moreover, one marker, SK05_27,742,506, was associated with four traits (ML, MT, LWR and MRW).

**Table 6:** Associations between KASP markers and phenotypes

KASP marker	Traits	*P* value	*R* ^2^	Chr.	Physical Position	Gene located or gene closely linked	Gene locus	Encoded protein	Known associated trait
SK05_27,742,506 #	ML	0.0067	0.1959	5	27,742,506	LTN1*	LOC_Os05g48390	ubiquitin conjugating enzyme protein	yield
	LWR	0.006	0.1928						
	MT	0.0096	0.0797						
	MRW	0.0053	0.2028						
SK02_1,968,779	ML	0.0067	0.1959	2	1,968,779	*	LOC_Os02g04450	plant-specific domain TIGR01589 family protein	
	LWR	0.006	0.1928						
	MT	0.0096	0.0797						
SK02_8,978,321	ML	0.0067	0.1959	2	8,978,321	*	LOC_Os02g15870	molybdenum cofactor biosynthesis protein 1	
	LWR	0.006	0.1928						
	ML	0.0096	0.0797						
SK03_15,001,183	ML	0.0067	0.1959	3	15,001,183	IRO3*	LOC_Os03g26210	helix-loop-helix DNA-binding domain containing protein	Green leafhopper resistance
	LWR	0.006	0.1928						
	MT	0.0096	0.0797						
SK03_16,075,626 #	ML	0.0067	0.1959	3	16,075,626	*	LOC_Os03g27990	STRUBBELIG-RECEPTOR FAMILY 7 precursor	Brown rice surface area
	LWR	0.006	0.1928						
	MT	0.0096	0.0797						
SK03_28,852,757	ML	0.0068	0.1961	3	28,852,757	*	LOC_Os03g50530	expressed protein	Alkali digestion value
	LWR	0.0061	0.1928						
	MT	0.0097	0.0804						
SK04_20,396,474	ML	0.0069	0.1965	4	20,396,474	–	–	Intergenic	
	LWR	0.0061	0.1926						
	MT	0.0094	0.0809						
SK04_25,898,378	ML	0.0053	0.2347	4	25,898,378	*	LOC_Os04g43750	kinase, pfkB family	
	LWR	0.0046	0.2383						
	MT	0.0078	0.0887						
SK06_26,266,828	ML	0.0069	0.1965	6	26,266,828	*	LOC_Os06g43650	secreted salivary protein, putative, expressed	
	LWR	0.0061	0.1926						
	MT	0.0094	0.0809						
SK09_3,717,701	ML	0.0067	0.1959	9	3,717,701	–	–	Intergenic	
	LWR	0.006	0.1928						
	MT	0.0096	0.0797						
SK03_15,397,382	MW	0.0081	0.1784	3	15,397,382	*	LOC_Os03g26920	OsSCP12	
	LWR	0.0077	0.1824						
SK04_22,435,296	ML	0.0036	0.2392	4	22,435,296	*	LOC_Os04g37730	RING-H2 finger protein ATL3B precursor	
	LWR	0.0092	0.1749						
SK05_5,396,755#	ML	0.0074	0.1985	5	5,396,755	*	LOC_Os05g09550	Der1-like family domain containing protein	grain width
	LWR	0.0066	0.1939						
SK05_5,537,852#	ML	0.0027	0.2493	5	5,537,852	*GW5*	LOC_Os05g09520	Calmodulin-binding motif family protein	grain width
	LWR	0.0046	0.2085						
SK06_7,670,859	ML	0.0074	0.1956	6	7,670,859	–	–	Intergenic	Alkali digestion value
	MRW	0.0067	0.1872						
SK09_15,765,355	ML	0.0075	0.1979	9	15,765,355	–	–	Intergenic	
	LWR	0.0073	0.1881						
SK06_2,781,588	MW	0.0067	0.1871	6	2,781,588	*OsSSI*	–	Intergenic	quality
	LWR	0.0066	0.191						
SK06_873,670	MW	0.0076	0.1908	6	873,670	*	LOC_Os06g02520	interacting protein of DMI3, putative, expressed	amylose content
	LWR	0.0025	0.26						
SK03_16,733,441#	ML	0.0038	0.2296	3	16,733,441	*GS3* ^*^	Os03g0407400	Transmembrane protein	grain shape
SK03_872,082#	ML	0.0091	0.1816	3	872,082	*	LOC_Os03g02440	WD repeat-containing protein 44, putative, expressed	number of spikelets per panicle
SK04_4,599,886	MT	0.0073	0.0958	4	4,599,886		LOC_Os04g08510	OsFBX117 - F-box domain containing protein, expressed	Green leafhopper resistance
SK05_5,339,085#	MW	0.0077	0.1796	5	5,339,085	GW5*	LOC_Os05g09500	Calmodulin-binding motif family protein	grain width
SK06_27,438,624#	LWR	0.0049	0.2072	6	27,438,624	*APO1* ^*^	LOC_Os06g45460	OsFBX202 - F-box domain containing protein	yield
SK06_27,481,339#	LWR	0.0048	0.2056	6	27,481,339	*APO1* ^*^	LOC_Os06g45460	OsFBX202 - F-box domain containing protein, expressed	yield
SK06_27,498,819#	LWR	0.0048	0.2056	6	27,498,819	*APO1*	LOC_Os06g45460	OsFBX202 - F-box domain containing protein	yield
SK06_27,526,542#	LWR	0.0048	0.2056	6	27,526,542	*APO1*	LOC_Os06g45460	OsFBX202 - F-box domain containing protein	yield
SK06_647,869	LWR	0.0073	0.1893	6	647,869	*	LOC_Os06g02160	AMP-binding enzyme family protein, expressed	Brown planthopper resistance
SK06_701,439	LWR	0.0088	0.194	6	701,439	*	LOC_Os06g02240	RNA recognition motif containing protein	Brown planthopper resistance
SK07_472,805#	ML	0.0021	0.2688	7	472,805	*DEP* ^*^	LOC_Os07g01820	OsMADS15	yield
SK08_21,582,840#	ML	0.0094	0.184	8	21,582,840	COE1*	LOC_Os08g34380	Receptor-like kinase, putative, expressed	yield
SK11_23,607,373	LWR	0.0084	0.1769	11	23,607,373	–	–	Intergenic	Green leafhopper resistance

# means the KASP marker was developed based on cloned genes or on identified loci responsible for yield; * means the KASP marker site is within the genomic sequence; − means the KASP marker is located within an intergenic region

Among the 31 markers, 13 were developed based on cloned genes or on identified loci related to yield traits. The R^2^ value of the 13 markers contributing to their corresponding traits ranged from 0.0797 to 0.2688. SK05_27,742,506 is located within the cloned gene *LTN1* sequence region and is significantly associated with four traits. The *LTN1* gene encodes a protein containing a ubiquitin-binding domain and can regulate grain shape by affecting the absorption of Fe, K, Na, Ca and other metal elements. SK03_16,075,626 was significantly associated with ML, the LWR and MT, which is consistent with previous GWAS results in which this locus was significantly correlated with seed surface area. SK03_16,075,626 is located within the LOC_Os03g27990 sequence, which encodes the precursor of STRUBBELIG-RECEPTOR FAMILY 7, whose function is still unknown. SK05_5,396,755 was significantly associated with ML and the LWR and has been reported in previous GWAS results. This marker was located within the LOC_Os05g09550 sequence region, which encodes Der1-like family domain-containing proteins. SK05_5,537,852 and SK05_5,339,085 were developed based on cloned *GW5* polymorphic loci. SK05_5,537,852 was significantly associated with ML and the LWR, while SK05_5,339,085 was significantly associated with MRW. SK03_16,733,441 is a FM of the *GS3* gene and is significantly associated with ML in this study. SK03_872,082, which is located within LOC_Os03g02440 and encodes a WD repeat-containing protein 44, was developed in accordance with previous GWAS reports and was significantly associated with ML. SK06_27,438,624, SK06_27,481,339, SK06_27,498,819 and SK06_27,526,542 were all developed based on the cloned gene *APO1*, which affects grain traits by regulating the formation of rice vascular bundles. These four markers were significantly associated with the LWR; SK06_27,481,339 is located within the *APO1* sequence interval. SK07_472,805 is located within the sequence of the cloned gene *DEP*, which encodes a transcription factor that consists of 267 amino acids, contains a MADS-box domain and plays an important role in the development of flower organs. SK08_21,582,840 is highly associated with ML and is located in *COE1*, which affects the growth of rice leaf veins and may indirectly affect grain development. Based on the above results, we detected cloned genes of *GS3* and *GW5*, and the markers that were developed from the cloned genes related to grain shape or from reported GWAS loci related to grain shape were verified in the present study. Therefore, markers related to important rice traits within core SNP arrays can be used for allele screening to provide useful information to breeders.

Notably, several KASP markers were found to be associated with milled grain shape and brown planthopper resistance simultaneously. Three markers, SK11_23,607,373, SK03_15,001,183 and SK04_4,599,886, were closely linked with the green leafhopper resistance genes *Grh2*, *Grh4* and *Grh6*(Fujita et al. 2010), respectively. Two other markers, SK06_647,869 and SK06_701,439, were closely linked with the brown planthopper resistance gene *BPH25(t)*(Fujita et al. 2010, Xiao et al. 2016). The discovery of these loci provides useful information for studying multiple effects of genes.

## Discussion

### Necessity and significance of developing core KASP-SNP markers

Molecular MAS plays an important role in rice molecular breeding. The quantity and detection methods of molecular markers play a pivotal role in the MAS procedure. SNP markers, which are third-generation molecular markers, will effectively push the development of molecular breeding because of their widespread distribution across genomes and their applicability for automatic detection. KASP detection technology, which is based on both PCR and fluorescence detection, can meet the requirements of low-, medium- and high-throughput genotyping, including that involving both SNP and InDel markers, and provides a flexible, relatively inexpensive solution for mass SNP molecular marker transformation and utilization. Compared with Sanger and high-throughput sequencing, KASP technology is more efficient at identifying polymorphic loci. The genotyping results of KASP and Sanger sequencing for identical loci were also compared in this study, and no differences were observed. A comparison of the KASP method and the allele-specific PCR (AS-PCR) method in studies of wild rice mutants revealed the specificity and sensitivity of the KASP assay (Rosas et al. 2014).

In this study, 596 loci were identified from several SNP datasets and were used to develop KASP markers. After removing some invalid loci, such as those that failed to amplify and displayed no polymorphism, we ultimately obtained 467 valid KASP markers, which constituted a core SNP array. The SNP conversion rate includes two types of rates: the design success rate and the work success rate. The ratio of SNP sites that can be used to design primers to the total number of SNP sites is called the design success rate. The work success rate refers to the number of SNP sites that can generate genotype calls via primers to the number of SNP sites with successful designed primers (Semagn et al. 2014, Fan et al. 2003, Hyten et al. 2010). A KASP-SNP marker set was reported by the IRRI (Pariasca-Tanaka et al. 2015). This set of markers was transformed from 2015 SNP loci, and the success rate for the locus primer design was 100%, which was higher than that in this study (94.8%); the work success rate of the SNP markers was 86.3%, which was lower than that in this study (93.62%). Compared with that in the IRRI study, the proportion of polymorphic markers in this study is much greater: 37% versus 83.69%, respectively. These differences may be because the IRRI markers were verified only in two rice materials, while our markers were identified in a large number of rice materials (481 accessions). Therefore, the proportion of detection results and degree of polymorphism were higher in our study than in IRRI study. The conversion success rate reached 93.6% in the present study; this percentage is similar to the 93–94% conversion rate reported by LGC Genomics (LGC Genomics applications note, http://www.kbioscience.co.uk/reagents/ KASP_Taqmancomparison.pdf). For maize, KBioscience successfully designed assays for 1250 SNPs that corresponded to 1536 SNPs from an Illumina GoldenGate chip (81.4%) (Semagn et al. 2014).

Compared with the reported SNP marker set, the core SNP markers in this study were derived from multiple rice SNP databases, e.g., the Gramene database (http://ensembl.gramene.org/genome_browser/index.html), the Rice Diversity Project database (https://www.ricediversity.org/), the Rice Genome Annotation Project database (http://rice.plantbiology.msu.edu/), and the Rice SNP-Seek Database(http://snp-seek.irri.org), as well as reported genes or trait-related loci. Therefore, the core SNP array carries more genetic information and is more comprehensive than the SNP marker set. Among the eight groups of molecular markers in the core SNP array, two groups comprise cloned genes that are tightly linked or are target markers: one group is the FMs, and the other group consists of reliable SNP markers associated with traits reported in previous GWASs. These traits provide valuable information for MAS. In addition to these trait-related markers, in the present study, SNP markers that were related to quality control and indica-indica groupings and that were highly polymorphic were developed, which are valuable for analyzing the genetic background of materials.

### Core SNP assays for rice germplasm assessment, genetic diversity and population evaluation

Genetic diversity acts as a reservoir for identifying superior alleles that control key agronomic and quality traits by allele mining (Nachimuthu et al. 2015). Assessment of genetic diversity and population structure in rice via different germplasm lines is often needed when developing core collections from national collections and international collections (Zhang et al. 2009, Zhang et al. 2011). Simple sequence repeat (SSR) markers are widely used in rice genetic diversity analyses because of their relatively large allelic variation, and SNP markers are also widely used in rice genetic diversity evaluation because of their easy automation. In this study, core SNP assays were also used for genetic diversity evaluation.

Based on the genotyping results of 467 core SNP markers, 481 rice germplasms were divided and categorized into three subgroups (two indica groups and one japonica group); this categorization seemed to differ from previously reported results in which only two groups, an indica group and a japonica group, were identified from collections (Zhang et al. 2009, Zhang et al. 2011). However, the *F*_*ST*_ value between the two indica subgroups was very low (0.0721). The average *F*_*ST*_ value between the two indica subgroups and the japonica subgroup was 0.489, which indicates that the indica population could be definitively distinguished from the japonica population; this value was similar to the *F*_*ST*_ value (0.517) of indica and japonica subspecies as determined via SNP markers (Muhamad et al. 2017). The genetic diversity and PIC of our population were 0.28 and 0.24, respectively. These results were similar to those of 215 rice germplasms, whose genetic diversity and PIC values were 0.26 and 0.24, respectively, and to those of 6984 rice varieties in northeastern India, whose genetic diversity and PIC values were 0.28 and 0.22, respectively, as determined via SSR markers (Xie et al. 2012, Choudhury et al. 2014). Based on the above results, we showed that the core SNP markers have a strong ability to reflect the genetic diversity of different materials.

To utilize the benefit from the functional KASP markers applied for our genotyping panel, molecular screening was carried out. Pariasca-Tanaka developed a KASP-SNP genotyping panel for detecting polymorphisms between different rice genera (Pariasca-Tanaka et al. 2015). KASP assays can also be used for genes that underpin key economic traits in crop breeding (Rasheed et al. 2016). For functional markers, KASP assays exhibit powerful detectability in breeding for disease resistance (Neelam et al. 2013). In our study, we used our developed core KASP assays to screen elite rice strains for superior quality. The genetic basis of rice in terms of eating and cooking quality is affected mainly by three physicochemical properties: AC, gel consistency (GC), and gelatinization temperature (GT); however, thus far, only the *Waxy* gene(ZY 1995) has been found to affect AC, and only the *ALK* gene (Gao et al. 2003, Gao et al. 2011) has been found to affect GT. Fragrance, which is controlled by *BADH2* located on chromosome 8S(Chen et al. 2008), is another popular quality sought by consumers worldwide. To screen elite rice strains for eating and quality trait improvement, three KASP assays developed from the above three genes were used for large-scale genotyping. Eighteen rice lines were successfully screened for subsequent breeding. This system provides a convenient and helpful method for excavating elite rice strains in breeding. Therefore, KASP assays offer a cost-effective and scalable flexibility to applications that require small to large numbers of markers, such as quality control analysis, marker-assisted recurrent selection, and marker-assisted backcrossing.

### The core SNP array provides a relevant method for excavating target traits

Candidate gene association mapping is used to detect functional SNPs or haplotypes associated with genes related agronomic traits so that identified germplasms carrying SNPs or haplotypes can be used for marker-assisted breeding(Huang and Brule-Babel 2012). In the core SNP array in the present study, 244 markers are related to important rice traits (yield, quality, biotic or abiotic stress, plant development and fertility). These markers provide important reference information for MAS-based breeding. To evaluate the validation of these trait-related markers, with milled grain properties as an example, a GWAS was conducted on 323 rice accessions via core SNP genotyping.

The marker SK05_5,396,755, which was significantly correlated with grain length and the LWR, was reported to be significantly correlated with grain width. The marker SK05_27,742,506 was significantly associated with grain length, the LWR and grain weight and was located within the cloned gene LTN1, which is a key functional gene related to yield (Hu et al. 2011). The markers SK05_5,537,852 and SK05_5,339,085, which were significantly correlated with grain length and the LWR, were reported to be within the sequence region of *GW5*, which is a functional gene responsible for grain width (Weng et al. 2008). SK06_27,481,339, SK06_27,438,624 and SK08_21,582,840, which were significantly correlated with milled rice length, were all located within the interval of the cloned yield-related gene; the corresponding cloned genes were *APO1* and *COE1*, respectively (Ikeda et al. 2007, Kitagawa et al. 2010). The marker SK03_16,075,626 was strongly associated with grain length, grain thickness and the LWR, which was consistent with previous GWAS-identified loci that partly contributed to grain surface parameters (Yang et al. 2014). The cloned gene *GS3* was identified via the FM SK03_16,733,441. The above results validated previous reports on grain traits, indicating the reliability and authenticity of the core SNP array. Furthermore, several KASP markers were found to be associated with milled grain shape and brown planthopper resistance simultaneously. Three markers, SK11_23,607,373, SK03_15,001,183 and SK04_4,599,886, were tightly linked to the green leafhopper resistance genes *Grh2*, *Grh4* and *Grh6* (Fujita et al. 2010), respectively. Two other markers, SK06_647,869 and SK06_701,439, were closely linked to the brown planthopper resistance gene *BPH25(t)*(Fujita et al. 2010, Xiao et al. 2016). The discovery of these loci provides useful information for studying multiple effects of genes. Marker-trait association analysis serves as a powerful tool for the dissection of complex agronomic traits and for the identification of alleles that can contribute to the enhancement of target traits (Yan et al. 2011). Therefore, core SNP markers related to important rice traits can be used for allele screening to provide useful information to breeders.

## Conclusion

In this study, we developed an efficient and versatile core SNP assays based on 467 KASP markers and successfully used them for rice germplasm assessment, genetic diversity and population evaluation. The core KASP arrays were also used for association analysis with milled grain traits. The results showed that this core SNP assays were effective and reliable, and will be valuable for promoting SNP molecular breeding in rice.

## Methods

### Plant material

A total of 530 rice accessions from various regions worldwide, including rice lines from the Germplasm Bank of South China Agricultural University, male-sterile lines, advanced breeding lines, and hybrid combinations, were used in this study (Additional file 2: Table S2). All accessions and lines were planted in the field in Guangzhou (a traditional flatland field), Guangdong Province, China, during the dry season (DS) in 2015 and during the wet season (WS) in 2016.

### Collection of SNPs

Several SNP datasets were used, including the Rice Diversity Project SNP dataset (http://www.ricediversity.org/), a high polymorphism information content (PIC) SNP dataset (Chen et al. 2011), a GWAS SNP dataset (Zhao et al. 2011), the SNP array from Affymetrix Rice 44 K SNP microarray (Kurokawa et al. 2016), the Rice Annotation Project Database (http://rapdb.dna.affrc.go.jp), the SNP array (Yonemaru et al. 2014) and the “OsSNPnks” 50 K SNP chip (Singh et al. 2015). Eight kinds of SNP data were chosen for developing KASP markers distributed throughout the genome (Table 1). Sixty-one SNPs of RiceOPA1.0 in subset 1 were used for quality control and for assigning accessions to subgroups (Additional file 3: Table S3). Considering the higher frequency of usage of indica rice, 64 SNPs from RiceOPA2.1 in subset 2, which were supposed to be effectively polymorphic between indica/indica populations, were specifically chosen (Additional file 4: Table S4). Fifty-three SNPs with a relatively high PIC from a set of breeder-friendly SNP markers were also added as subset 3 of our genotyping panel (Additional file 5: Table S5). Functional nucleotide polymorphism (FNP) has been useful for selection in rice breeding, and 21 thoroughly studied FNP markers were selected; these markers composed subset 4 (Additional file 6: Table S6). Subset 5 (Additional file 7: Table S7) and subset 6 (Additional file 8: Table S8) comprised reported key SNP-targeted genes or overlapping gene intervals, which were designed for gene tracking. SNPs associated with important traits such as flowering time, blast resistance, amylose content (AC), and protein content revealed by GWAS via cross-population-based mapping were introduced as subset 7 (Additional file 9: Table S9). The trait-associated SNPs were selected based on their correlation degree with the agronomic traits. Only SNPs offering great contribution to phenotypic variance with significant *P* values were included in subset 7. Based on the SNP distribution, randomly selected markers for large gaps in the chromosomes were supplemented for gap filling in subset 8 (Additional file 10: Table S10). A total of 596 SNP sites were chosen for primer design and genotyping.

### Genomic DNA extraction and SNP genotyping

The rice genomic DNA was extracted using the cetyl-trimethylammonium bromide (CTAB) method and quantified using a NanoDrop ND-1000 spectrophotometer (Thermo Scientific, Wilmington, USA). To increase the efficiency and reduce the genotyping costs, high-throughput DNA extraction method (Chen et al. 2016) was applied for rapid DNA extraction of batch samples. The selected SNPs were sent to LGC Limited, UK, for primer design. For these SNPs, we extracted 250 bp surrounding the candidate SNP on either side and ordered KASP primer oligos from LGC. The genotyping assays were tested in a 96-well format and established as 10 μL reactions (4.85 μL of template DNA (50–75 ng), 5.0 μL of 2× Kaspar mix, and 0.15 μL of primer mix). PCR was performed on a StepOne Plus machine in accordance with the following protocol: preread stage at 30 °C for 1 min, hot start at 95 °C for 15 min, 10 touchdown cycles (95 °C for 20 s; touchdown at 65 °C, − 1 °C per cycle, 25 s) and then 26 cycles of amplification (95 °C for 10 s; 57 °C for 60 s).

Fluorescent data were collected during the preread and postread stages (30 °C for 1 min). For most of breeders in labs, 96-SNP array is the most commonly used because it is more economic, operable and use-friendly for various-sample-size genotyping. Actually KASP assays can also be manipulated on 384-plate format. The 384-plate format PCR system can be established as 5 μL reactions (2.5 μL of template DNA (25 ng), 2.5 μL of 2× Kaspar mix, and 0.07 μL of primer mix). The genotyping efficiency can be more greatly upgraded with the 384-plate format and the genotyping cost can be reduced at the same time due to less reagent usage per assay. The breeders can adjust the genotyping system according to specific needs and laboratory equipment conditions. Once the run was completed, analysis was carried out with StepOne Software V2. The primers of all the KASP arrays used in our study can be viewed in Additional file 11: Table S11.

### Statistical analyses and marker-trait association studies

The SNP allele frequency and PIC were estimated for each locus using PowerMarker V3.25(Liu and Muse 2005). In addition, genetic distances between genotypes and neighbor-joining (NJ) tree distances were calculated using Power Marker 3.5. Genetic similarity coefficients between each variety were calculated using NTSYS-pc 2.10. A similarity matrix was used to construct a dendrogram via the unweighted pair-group method arithmetic average (UPGMA) algorithm and nested clustering (SHAN) routine to determine the genetic relationships among the materials. The UPGMA trees were visualized with MEGA 5(Tamura et al. 2011). The number of alleles per locus, the diversity index, and the PIC were subsequently estimated. The population structure was estimated with the program Structure V2.3.2 (Falush et al. 2003). The hypotheses for 2–10 subpopulations (K) with an admixture model and correlated allelic frequencies were tested; the length of the burn-in period was equal to 10,000 iterations, and 100,000 Markov Chain Monte Carlo replications were performed after the burn-in period. Ten runs of the Structure program were performed, and an average likelihood value, LnP(D), across all runs was calculated for each K. The most likely number of clusters (K) was selected by comparing the logarithmized probabilities of the LnP(D) and ΔK data.

The milled rice shape parameters of 323 out of 481 germplasms were investigated. Images of the more than 100 milled rice grains were captured via a CanoScan 5600F scanner (Canon, Japan) and the supplied software; the images were not enhanced. The length, width, length/width ratio (LWR), thickness, and 100 milled rice weight were measured precisely using SmartGrain software (Tanabata et al. 2012). An association study between marker alleles and the above trait data was performed with TASSEL 5.0 software, taking into account gross level population structure (Q) (Bradbury et al. 2007). The *P* value (marker) determining whether a marker is associated with a trait and the *R*^2^ value (marker) indicating the fraction of the total variation explained by the marker were reported.

## Additional files

Additional file 1: **Table S1.** Primers for sequencing and genotyping. (XLSX 10 kb)

Additional file 2: **Table S2.** List of the rice germplasm used in this study. (XLSX 39 kb)

Additional file 3: **Table S3.** Subset 1 of SNPs from Kurokawa et al. (2016). (XLSX 12 kb)

Additional file 4: **Table S4.** Subset 2 of SNPs from Kurokawa et al. (2016). (XLSX 13 kb)

Additional file 5: **Table S5.** Subset 3 of SNPs. (XLSX 12 kb)

Additional file 6: **Table S6.** Subset 4 of SNPs. (XLSX 11 kb)

Additional file 7: **Table S7.** Subset 5 of SNPs. (XLSX 14 kb)

Additional file 8: **Table S8.** Subset 6 of SNPs. (XLSX 25 kb)

Additional file 9: **Table S9.** Subset 7 of SNPs. (XLSX 12 kb)

Additional file 10: **Table S10.** Subset 8 of SNPs from Zhao et al. (2011) & Yonemaru et al. (2014). (XLSX 12 kb)

Additional file 11: **Table S11.** KASP assay primers used in the study. (XLSX 39 kb)
